# Draft Genome Sequence of *Serratia grimesii* Strain A2

**DOI:** 10.1128/genomeA.00937-14

**Published:** 2014-09-18

**Authors:** Ayslu M. Mardanova, Anna A. Toymentseva, Adeliya G. Gilyazeva, Sergey V. Kazakov, Elena I. Shagimardanova, Sofia Yu Khaitlina, Margarita R. Sharipova

**Affiliations:** a Institute of Fundamental Medicine and Biology, Kazan (Volga region) Federal University, Kazan, Russia; b Computer Technologies Laboratory, Information Technologies, Mechanics & Optics University, Saint Petersburg, Russia; c Institute of Cytology Russian Academy of Science, Saint Petersburg, Russia

## Abstract

We report the first draft genome assembly of *Serratia grimesii* strain A2, previously identified as *Escherichia coli* strain A2, which produces protease ECP32 with a high specificity toward actin. *S. grimesii* strain A2 has multidrug resistance associated with a number of efflux pump genes.

## GENOME ANNOUNCEMENT

A Gram-negative bacterium from the family *Enterobacteriaceae* that produces a new metalloproteinase, ECP32, with a high specificity toward actin was previously isolated (1–3). The bacterium was identified as atypical strain *Escherichia coli* A2 (1, 2). With the use of the Vitek 2 system (bioMérieux, France) and partial sequencing of the 16S rRNA gene, the strain А2 was reidentified as *Serratia grimesii* A2 (4). *S. grimesii* was classified as a part of the *Serratia liguefaciens* complex, along with *Serratia proteamaculans* (5). It was shown that ECP32 metalloprotease is homologous to actin-specific grimelysin from *S. grimesii* DSMZ30063 (4). Protease ECP32/grimelysin was successfully used in studies of actin structure/function relationships and mechanisms of actin polymerization (6, 7). Moreover, it was shown that introduction of the ECP32 protease gene to nonpathogenic *E. coli* results in acquisition of the ability to invade eukaryotic cells (8).

*S. grimesii* A2 genomic DNA, isolated using the GeneJET genomic DNA purification kit (Fermentas, Lithuania), was whole-genome shotgun sequenced using the 454 GS Junior (Roche) platform. The sequencing resulted in 5,137,381 high-quality reads with an average read length of 500 bp and total 12× coverage. Testing different *de novo* assemblers (GS Junior, Roche; Geneious R7; SPAdes 3.0; ABySS 1.3.2; and MIRA 3-9-17), we applied the SPAdes platform (http://bioinf.spbau.ru/en/spades) and obtained 149 contigs (99 contigs larger than 500 bp; *N*_50_, 108,561 bp). The chromosome has an overall G+C content of 52.85%. Nontranslated genes were predicted using tRNAscan-SE (9) and RNAmmer (10), which identified 69 tRNAs and 11 rRNAs. Open reading frame (ORF) prediction and annotation were performed through the Rapid Annotation using Subsystems Technology (RAST) pipeline (11). The functional comparison of genome sequences available on the RAST server revealed that the closest neighbors of *S. grimesii* A2 are *S. proteamaculans* 568 (score 525) followed by *Serratia plymutica* A30 (score 493), *Serratia odorifera* 4Rx13 (score 489), and *Serratia marcescens* Db11 (score 462). A total of 5,006 protein-coding sequences (CDSs) were identified, among which 2,883 CDSs (58% of the genome) were assigned to 1 of the 579 RAST subsystems. The annotated genome has 126 genes of the virulence, disease, and defense subsystem, among which 93 genes were predicted as antibiotic resistance genes. Thus, the *S. grimesii* A2 strain possesses multidrug resistance, and it is probably associated with a number of efflux pump genes.

### Nucleotide sequence accession numbers.

The *S. grimesii* A2 whole-genome shotgun project has been deposited in DDBJ/ENA/GenBank under the accession no. JGVP00000000. The version described in this paper is the first version.
